# Genotypic variation in spike fertility traits and ovary size as determinants of floret and grain survival rate in wheat

**DOI:** 10.1093/jxb/erw200

**Published:** 2016-06-08

**Authors:** Zifeng Guo, Gustavo A Slafer, Thorsten Schnurbusch

**Affiliations:** ^1^HEISENBERG-Research Group Plant Architecture, Leibniz Institute of Plant Genetics and Crop Plant Research (IPK), Corrensstr. 3, 06466 Stadt Seeland, OT Gatersleben, Germany; ^2^ICREA (Catalonian Institution for Research and Advanced Studies), Department of Crop and Forest Sciences and AGROTECNIO (Centre for Research in Agrotechnology), University of Lleida, Av. Rovira Roure 191, 25198 Lleida, Spain

**Keywords:** Fertile florets, floret abortion, fruiting efficiency, grain number.

## Abstract

Spike fertility traits are critical attributes for grain yield in wheat (*Triticum aestivum* L.). Here, we examine the genotypic variation in three important traits: maximum number of floret primordia, number of fertile florets, and number of grains. We determine their relationship in determining spike fertility in 30 genotypes grown under two contrasting conditions: field and greenhouse. The maximum number of floret primordia per spikelet (MFS), fertile florets per spikelet (FFS), and number of grains per spikelet (GS) not only exhibited large genotypic variation in both growth conditions and across all spikelet positions studied, but also displayed moderate levels of heritability. FFS was closely associated with floret survival and only weakly related to MFS. We also found that the post-anthesis process of grain set/abortion was important in determining genotypic variation in GS; an increase in GS was mainly associated with improved grain survival. Ovary size at anthesis was associated with both floret survival (pre-anthesis) and grain survival (post-anthesis), and was thus believed to ‘connect’ the two traits. In this work, proximal florets (i.e. the first three florets from the base of a spikelet: F1, F2, and F3) produced fertile florets and set grains in most cases. The ovary size of more distal florets (F4 and beyond) seemed to act as a decisive factor for grain setting and effectively reflected pre-anthesis floret development. In both growth conditions, GS positively correlated with ovary size of florets in the distal position (F4), suggesting that assimilates allocated to distal florets may play a critical role in regulating grain set.

## Highlight

Outcomes of floret initiation, mortality/survival, grain set/abortion, and fruiting efficiency have been quantified in 30 cultivars and connected to the processes determining the fate of floret primordia using ovary size.

## Introduction

Raising wheat (*Triticum aestivum* L.) yield remains one of the main objectives of wheat breeding efforts (Dencic *et al.*, 2000; Kirigwi *et al.*, 2007; Edgerton, 2009; Shewry, 2009; Ainsworth and Ort, 2010). Because yield is a complex, multifactorial trait (Reynolds *et al.*, 2009; Foulkes *et al.*, 2011; Parry *et al.*, 2011), continually achieving this aim has become increasingly difficult (Reynolds *et al.*, 2012). The better we understand the genetic factors determining yield, the more likely we are to effect relatively large rates of genetic gains in yield (Slafer, 2003). Grain yield in wheat is commonly reported to be associated with grain number (Fischer, 2008; Lizana and Calderini, 2013), and seems impossible to achieve large gains in yield without increasing grain number (Slafer *et al.*, 2014). In this context, improving spike fertility seems critical (Foulkes *et al.*, 2011; Reynolds *et al.*, 2012). Most of what we know of spike fertility comes from studies analysing genetic and environmental factors that influence spike dry weight at anthesis (AN) and fruiting efficiency (FE) (Slafer *et al.*, 2015a). Although this approach has been extremely useful (Fischer, 2011), it considers only the final number of grains set, focusing on the final output of the reproductive biology process determining spike fertility.

**Fig. 1.**
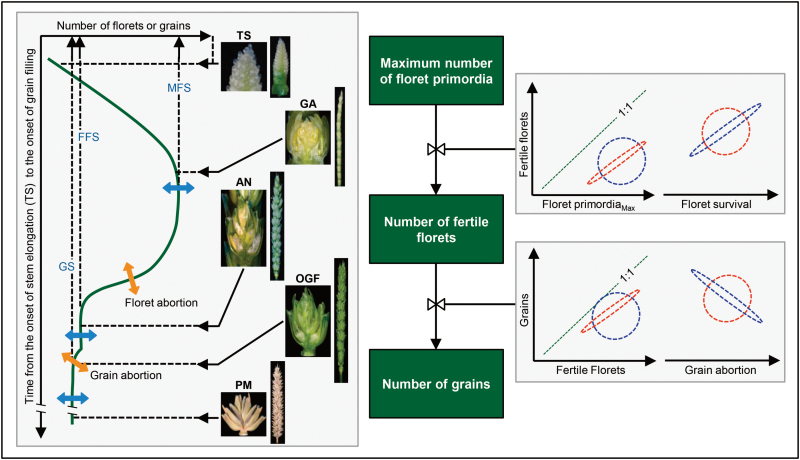
Schematic diagram describing the determination of spike fertility in wheat. In the left part of the scheme the dynamics of floret generation/degeneration and grain set through different stages of development (TS: terminal spikelet; GA: green anther; AN: anthesis; OGF: onset of grain filling; PM: physiological maturity) is illustrated, with blue arrows indicating possible genotypic variation in the state variables and orange arrows indicating possible genotypic variation in the processes of floret survival and grain abortion (pictures displaying individual floret/grain and whole spike morphology at each stage are not to scale). On the right is a simple flow diagram showing the key determinants of the three critical state variables in the generation/degeneration of organs resulting in spike fertility (maximum number of floret primordia, number of fertile florets, and number of grains): the number of fertile florets may be a consequence of the maximum number of floret primordia (hypothetical relationships are illustrated in red) or the level of floret survival (hypothetical relationships in blue); while the number of grains may be a consequence of the number of fertile florets (hypothetical relationships are illustrated in red) or the level of grain abortion (hypothetical relationships in blue). We have used examples of two different regression trends between survived number (fertile florets and grains), survival (floret and grain survival), and abortion (floret and grain abortion).

An alternative/complementary approach to understand the grain number and spike fertility is to consider the complete process of reproductive biology that culminates in grain number (Fig. 1). First, a large number of floret primordia are initiated in each of the spikelets of the spike from approximately the terminal spikelet stage (TS) to around the stage of green anthers (GA), when the maximum number of floret primordia per spikelet (MFS) and per spike is frequently reached (Guo and Schnurbusch, 2015). Many (usually most) of the primordia do not continue to develop normally (that is, they die) and only a relatively small fraction of the initiated floret primordia survive to produce a number of fertile florets per spikelet (FFS) and per spike at AN. The ovaries of these florets are then fertilized and, during the lag phase occurring immediately after AN, a proportion (variable, though normally small) of these fertilized ovaries abort and the rest set grains, thereby determining yield at maturity. Grain number is therefore the outcome of floret primordia initiation and survival during the stem elongation phase, which produces fertile florets at AN and grain setting immediately after AN (Slafer *et al.*, 2015b). The rate of floret survival is generally low (Miralles *et al.*, 1998a; Miralles *et al.*, 1998b; Ghiglione *et al.*, 2008; Gonzalez *et al.*, 2011a; Ferrante *et al.*, 2013a); therefore, there might be great potential for improving grain yield in wheat by increasing the rate of floret survival (Sreenivasulu and Schnurbusch, 2012).

A widely accepted hypothesis supported by previous studies is that the variation in grain number is associated with changes in the availability of assimilates during the period of stem elongation, when floret survival takes place (Siddique *et al.*, 1989; Slafer and Andrade, 1993). Thus, wheat spikes overproduce energetically inexpensive floret primordia and, when floret development requires increasing amounts of resources, the number of primordia that become fertile florets is adjusted to the actual assimilate availability (Sadras and Slafer, 2012). This seems in line with recent evidence that the loss of floret primordia (determining the rate of floret survival) is resource driven (Gonzalez *et al.*, 2011b; Ferrante *et al.*, 2013b). For instance, dwarfing genes have been found to increase spike fertility, due to reduced stem growth allowing for more assimilate translocation to the spike (Miralles *et al.*, 1998b). Similarly, a longer duration of stem elongation allowed greater assimilate translocation to spike growth, increasing the FFS at AN in wheat (Gonzalez *et al.*, 2003) and number of fertile spikelets in barley (*Hordeum vulgare*) (Alqudah and Schnurbusch, 2014); likewise, detillering plants, which allows more resources to become available for the main shoot spike, also increases FFS (Ferrante *et al.*, 2013a; Guo and Schnurbusch, 2015). Increased plant density reduces both the number of floret primordia initiated and floret survival, resulting in the typical agronomic reaction of reduced individual spike fertility in response to increased density. A study analysing this issue in more detail found that a low red/far-red ratio (simulating dense stands) can reduce spike fertility due to a delayed spike growth and results in fewer floret primordia initiated and fewer fertile florets (Ugarte *et al.*, 2010). High nitrogen levels seem to accelerate developmental rates of floret primordia by increasing spike growth (though not consistently altering phasic development; Hall *et al.*, 2014), allowing an increase in the number of fertile florets and in grain setting in durum wheat (Ferrante *et al.*, 2010, 2013*a*) and barley (Arisnabarreta and Miralles, 2010). Complementarily, shading treatments immediately before AN (during the period of floret mortality) significantly reduced spike fertility (Fischer and Stockman, 1980; Slafer and Savin, 1991), irrespective of the yield potential of the genotype (Slafer *et al.*, 1994). High temperatures (up to 30°C) during the pre-AN phase, especially from booting to AN, can result in a considerable reduction in the number of fertile florets at AN, possibly affecting sensitive stages of pollen meiosis (Saini *et al.*, 1983, 1984; Dawson and Wardlaw, 1989), but also due to a reduction in assimilate availability for floret survival. The latter is evidenced by the fact that moderately high temperatures (not damaging pollen viability) also reduce spike fertility by shortening the duration of stem elongation and consequently reducing assimilate availability per unit of developmental time (Fischer, 1985; Ugarte *et al.*, 2007). High temperatures may also increase grain abortion (Prasad and Djanaguiraman, 2014).

Most of the few studies analysing the dynamics of floret development as a determinant of grain number have focused on environmental effects. Very few have included genotypic variation, and, when that variation was considered, the number of genotypes analysed was extremely low. Assuming a parallelism with the relationships uncovered with environmental effects (an analogy that may not be strictly correct; Slafer *et al.*, 2014), it might be hypothesized that genetic differences in spike fertility are based on differences in floret survival (represented by the red dotted curves in the models on the right of Fig. 1). To the best of our knowledge, only Gonzalez-Navarro *et al.* (2015) analysed the dynamics of floret development with a reasonable number of genotypes, and their conclusions provided preliminary support to the first part of this hypothesis (i.e. genotypic variation in fertile florets was more related to variation in floret survival than in maximum number of floret primordia; Gonzalez-Navarro *et al.*, 2015). Differences between genotypes must be uncovered and quantified for the selection of prospective parents in crosses aimed to further improve yield potential. Genotypes that can be readily used in breeding programmes aiming to increase yield potential (for which breeders pyramided genes during many generations) include virtually only elite material, like commercial cultivars. In this study, we aimed to analyse genotypic variation in grain number by analysing not only the maximum number of floret primordia and floret survival to produce fertile florets but also grain abortion (i.e. failure of fertile florets to set grains) under two contrasting environmental conditions. We expanded considerably the genotypic variation explored so far by trialling 30 cultivars that showed large genotypic variation for thermal time (°Cd), absolute growing time (days), and floral organ development (Guo *et al.*, 2015).

## Materials and methods

### Plant material and growth conditions

Experiments were carried out at the Leibniz Institute of Plant Genetics and Crop Plant Research (IPK), Gatersleben, Germany (51° 49′ 23″ N, 11° 17′ 13″ E, altitude 112 m) during the 2012/13 growing season in two contrasting environmental conditions: greenhouse and field. Thirty European hexaploid winter wheat cultivars were grown, including 23 photoperiod-sensitive and 7 photoperiod-insensitive cultivars. These genotypes can also be classified into 24 semi-dwarf and 6 tall cultivars. Marker information for all 30 cultivars is presented in Supplementary Table S1. Forty plants per cultivar were planted in each of the growth conditions (a total of 2400 plants). Growth conditions for both experiments have previously been described (Guo *et al.* 2015); they matched in terms of temperature, day length, planting date, and density.

### Phenotypic staging and measurements

To determine the maximum number of floret primordia, plants around the GA stage (glumes cover all but the tips of florets; Kirby and Appleyard, 1987) were phenotyped (Guo and Schnurbusch, 2015). Every cultivar was examined every 2–3 days under a stereomicroscope (Stemi 2000-c, Carl Zeiss Micro Imaging GmbH, Göttingen, Germany). Samples were taken at AN when yellow anthers extruded and became visible in F1 and F2 florets. After the dissection of the floral developmental process, we confirmed the previous finding that MFS consistently occurred at around GA stage (Guo *et al.*, 2015). FFS and GS were obtained at AN and physiological maturity (PM), respectively. Compared with fertile florets, aborted florets are dry and transparent. The clear distinction between fertile and aborted florets can be seen in figure 4 of Guo and Schnurbusch (2015). We defined fertile florets as those that reached stage W10 of the Waddington scale as in a previous study (Ferrante *et al.*, 2010). Ideally, one should follow the stages of development of the selected ‘fertile’ florets until a few days after the most proximal florets are in W10, to make sure all the florets are fertile. Owing to the high number of genotypes and treatments in our study, this was not possible. As a compromise, we determined the number of fertile florets at AN (i.e. anthers extruded at F1 and F2), and then considered F3 and F4 to be ‘fertile florets’ if they were at that time in stages later than W8, as once florets get to stage W8 they can be considered irreversibly committed to producing a fertile floret (accepting that this is stage W10, a scale focused on carpel development), although it is possible for a fertile floret to abort after AN due to its size and/or its delayed condition. (In papers reporting the dynamics of floret development it is possible to see cases in which primordia may stop developing [and die] between W8 and W10, but it is only because the reported cases are averages of several florets measured for each particular treatment. Individually seen, it is virtually universal that florets developing beyond W8 would reach W10, even if few days later than AN of F1 and F2.) Even if a floret does not have viable pollen (male sterility), it can still be considered fertile if it reaches W10 because if it were fertilized with pollen from other florets it would produce a grain (and cross-pollination may be more likely with pollen from other florets).

**Fig. 2.**
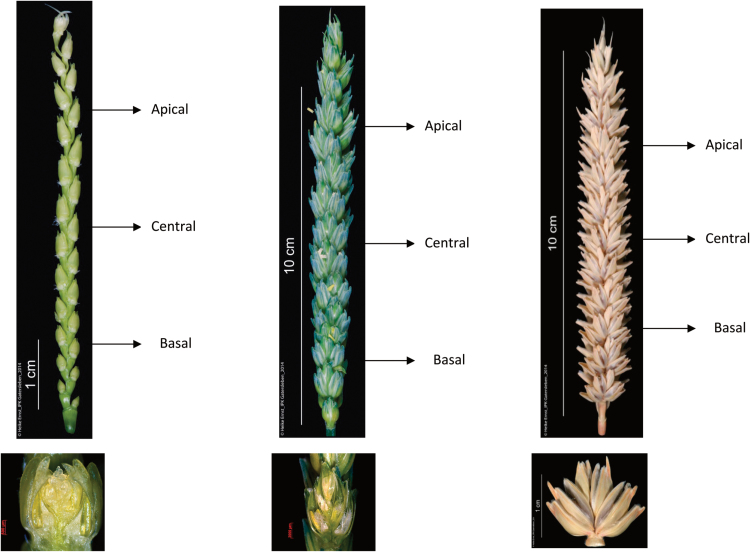
The apical, central, and basal spikelets for the measurements of the maximum number of floret primordia per spikelet at GA stage, number of fertile florets per spikelet at AN, and number of final grains per spikelet at PM (Guo and Schnurbusch, 2015).

**Fig. 3.**
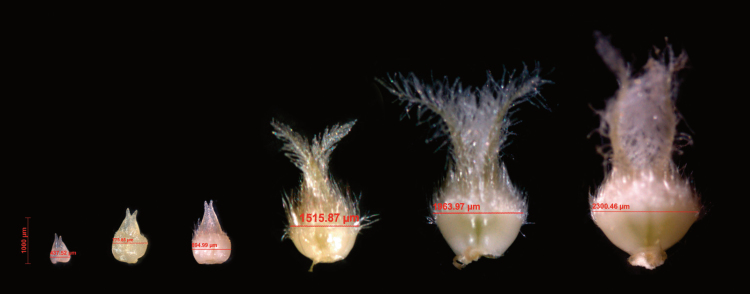
Wheat ovary growth and development over time. This figure shows the development of the ovary from early stages to AN and how ovary size was determined in this study. The first two ovaries are from F4 and F1 at GA stage, the third one is from F3 at yellow anther stage (this stage was defined in Guo and Schnurbusch, 2015), the fourth and fifth are from F3 and F1 at the heading stage (the stage was defined in Guo and Schnurbusch, 2015), the last ovary is from F1 at AN.

Three plants from each cultivar were randomly selected for phenotypic measurements at GA and AN, and six plants at PM. The MFS, FFS, and GS were measured at three positions on the spikes from the main culms: apical (the third spikelet from the top), central (the spikelet in the centre of the spike), and basal positions (the third spikelet from the bottom) (Fig. 2). At AN, spikelets from the centre of the main culm spike were dissected to obtain digital images of ovaries of the first (F1), third (F3), and fourth (F4) florets from the base of the spikelet; when the ovary was degenerated, the size was not measured. Ovary size was recorded as ovary width as indicated in Fig. 3. Ovary width was measured using a stereomicroscope and Carl Zeiss Imaging System version AxioVision Rel. 4.8.2. There are several reasons why we chose to examine florets in positions F1, F3, and F4. First, the ovary in the F1 position develops early and is stable; second, ovary size at F2 is similar to F1, so we expected similar results; finally, the F3 and F4 positions are important because these are most frequently the vulnerable floret primordia determining the final number of fertile florets. The spike and main culm were separately dried in two cellophane bags at 60°C for 3–5 days for dry weight measurement. Stem dry weight refers to the dry weight of one main shoot culm, including leaves but without spikes.

Floret survival was calculated as the proportion of the maximum number of floret primordia that reached the stage of fertile florets (FFS MFS^−1^), and grain survival was determined as the proportion of the fertile floret number that set normal grain (GS FFS^−1^). Floret abortion was determined as the number of floret primordia that did not reach the stage of fertile florets, and grain abortion was calculated as the number of fertile florets that did not produce a normal grain. ‘Normal grains’ are defined as seeds that are completely developed, are not shrivelled, and have a size that is not particularly reduced.

Statistical analysis, including ANOVA and determination of heritability, have been described in previous work (Guo *et al.*, 2015).

## Results

### Genotypic variation in, and stability of, floret fertility-related traits

**Fig. 4.**
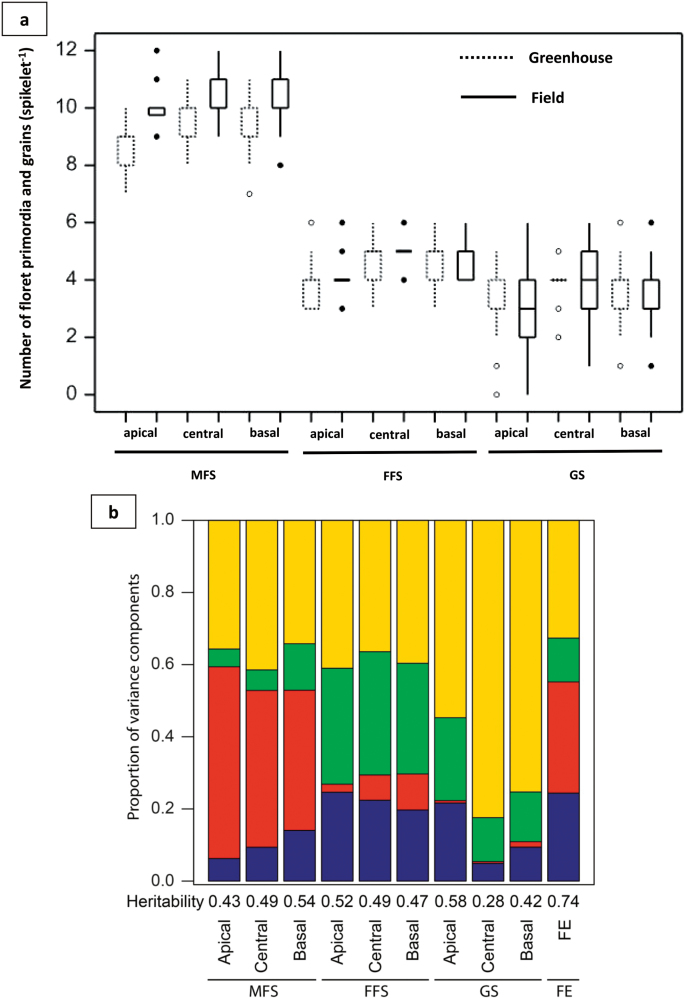
Genotypic variation of spike fertility traits. (**a**) Range of maximum number of floret primordia (MFS), number of fertile florets (FFS), and number of grains (GS) per spikelet in the apical, central, and basal spikelets of the spikes (see Fig. 2) in two growth conditions. For a few results within FFS and GS, the values were identical, therefore they are displayed as a line (not a box). (**b**) Proportions of variance components of MFS, FFS, GS, and fruiting efficiency (FE). The numbers on the *x*-axis represent the broad sense heritability of the corresponding traits.

MFS, FFS, and GS varied widely in both growth conditions and across all spikelet positions studied (Fig. 4a). Despite these strong variations, the ranges of the three traits at the apical, central, and basal spikelets of the spike were identical between all the genotypes and showed a reasonable degree of consistency between the two different growth conditions, as well as relatively high heritability (Fig. 4b). This indicates a common genetic base for the improvement of floret fertility and wheat breeding based on these traits. In addition, MFS displayed a high environmental sensitivity while GS showed large variation within individual genotypes (Fig. 4b). FE (here defined as grains set per unit chaff weight, i.e. the non-grain spike dry weight at physiological maturity) also displayed strong genotypic variation across the 30 genotypes, but there was no significant difference between greenhouse and field conditions, indicating that FE is consistent across variable conditions (Supplementary Fig. S1). The broad genotypic variation (Supplementary Fig. S1) and relatively high heritability (Fig. 4b) indicate the large potential and a genetic basis for the increase in FE. Because the environmental effects (σ^2^_E_) and influence of the interaction between environment and genotype (σ^2^_G:E_) were large for most traits according to an ANOVA analysis (Fig. 4b), the regression values for most traits between greenhouse and field conditions were low (Supplementary Fig. S2). In summary, there was a large degree of variation and also moderate to high heritability among the 30 cultivars analysed for all the traits determined; detailed data for individual genotypes are shown in Supplementary Tables S2, S3, S4, and S5.

### Importance of differences in MFS, floret abortion, and floret survival in determining genotypic differences in FFS

**Fig. 5.**
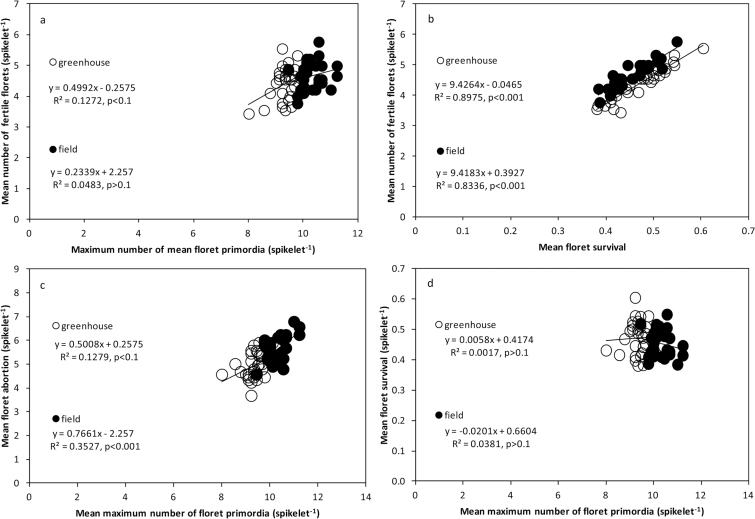
(**a–d**) Relationships between mean maximum number of floret primordia, mean number of fertile florets, mean floret survival, and mean floret abortion within spikelets under field and greenhouse conditions. All the traits are displayed as averages of the apical, central, and basal spikelets.

Averaging across spikelets, it is quite clear that even though there was substantial phenotypic variation in MFS (average of the three spikelets), the genotypic differences in FFS (average of the three spikelets) were almost exclusively due to genotypic differences in the survival of floret primordia (average of the three spikelets) (Fig. 5a, b). This overwhelming importance of floret primordia survival compared with MFS as the main determinant of genotypic differences in FFS was strongly consistent across the two contrasting environments (Fig. 5b). The fact that FFS was virtually unrelated to MFS was at least partly due to the fact that the larger the number of floret primordia initiated, the higher the tendency for primordia to be aborted (Fig. 5c). Part of the irrelevance of the differences in MFS between the 30 cultivars analysed in determining genotypic differences in FFS was due to the fact that there was no relationship between these two determinants (MFS and floret survival) of FFS (Fig. 5d), and that the relative variation in floret survival (~50% difference from <0.4 to ~0.55–0.6) was much higher than that in MFS (~20%, from 8–10 primordia in greenhouse or from 9–11 in the field). Analysing the results at the individual spikelet position level, the relationships between MFS and floret survival were weak in most cases (Supplementary Fig. S3), although there were a number of exceptions (although in these exceptions the relationships were significant, the values of R^2^ were small).

We found compelling evidence for the relative importance of floret survival compared with MFS in determining genotypic differences in FFS among modern cultivars after analysing the relationships for each spikelet position independently. At each particular spikelet position, and in both growth conditions, FFS was strongly significantly related to the proportion of primordia surviving (Supplementary Fig. S4a, b, c) and unrelated to MFS (Fig. S4d, e, f). Again, at each spikelet position, it was evident that differences in MFS significantly induced parallel differences in floret abortion (Supplementary Fig. S4g, h, i). While the relationship between FFS and floret survival was highly significant for all three spikelet positions in both field and greenhouse conditions (R^2^ ranging between 0.76 and 0.89; *P* < 0.001 in all six cases), the relationship between FFS and MFS was only significant in three of the six cases analysed. Even in those in which it was significant, R^2^ was very small (MFS never explained more than 18% of the variation in FFS).

### Importance of differences in FFS and grain abortion in determining genotypic differences in GS

**Fig. 6.**
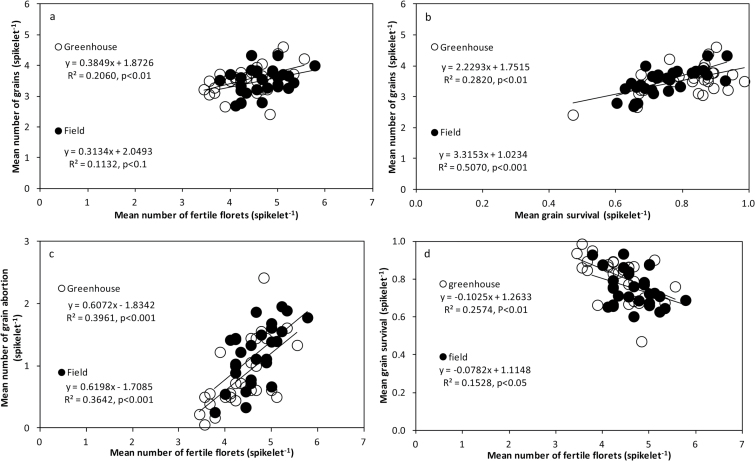
(**a–d**) Relationships between mean maximum number of fertile florets, mean number of grains, and mean grain abortion per spikelet under field and greenhouse conditions. All the traits are displayed as averages of the apical, central, and basal spikelets.

Consistent across both environments, genotypic differences in GS were positively related to those in FFS (Fig. 6a). However, even though R^2^ was statistically significant, the proportion of the variation explained was moderate. This was because GS was also related to the likelihood of a fertile floret to set a grain (Fig. 6b). In general, genotypes producing more FFS presented higher grain abortion (Fig. 6c) and lower grain survival (Fig. 6d), though the absolute differences in FFS overrode those in grain abortion.

Analysing the results at the individual spikelet position level showed that the relationships between GS and grain abortion were maintained (Supplementary Fig. S5a, b, c), while those between GS and FFS were maintained in the greenhouse experiment but disappeared in apical and central spikelets (and were maintained only in basal spikelets) in the field experiment (Supplementary Fig. S5e, f, g). The positive relationship (with slopes consistently smaller than 1) was also maintained between grain abortion and FFS at the spikelet level position (Supplementary Fig. S5h, i, j).

### Genotypic variation in spike fertility and size of the ovaries

**Fig. 7.**
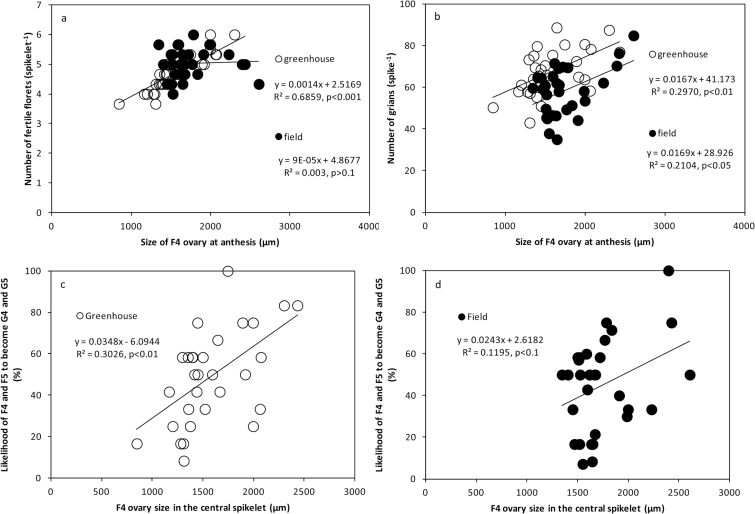
Associations of F4 (floret 4, the fourth floret from the base of the spikelet) ovary size in the central spikelet with FFS, GS, and the likelihood of F4 and F5 fertile florets becoming grains G4 and G5 (grains 4 and 5, the fourth and fifth grains from the base of spikelet) under field and greenhouse conditions. (**a, b**) Association between ovary size and number of fertile florets and grains, which can connect the pre-AN process (fertile florets) and post-AN (grains). (**c**, **d**) The likelihood of F4 becoming G4 and F5 becoming G5 is considered to be 100% (F4-G4, 0–50%; F5-G5, 51–100%). In a previous publication, a close relationship was found between ovary size at different positions (Guo *et al.*, 2015). Hence, although we did not measure ovary size at the F5 position (generally it is too small to be measured), F4 ovary size also indicates the likelihood of grain setting at the F5 position. Here, if the likelihood is above 50%, it means that the F5 is also likely to set grain.

Proximal florets became fertile in almost all spikelets and therefore differences in spike fertility depend on the likelihood of distal florets surviving to produce a fertile floret at, and set a grain immediately after, AN. It therefore seems relevant to determine the dependence/independence of phenotypic differences in spike fertility and the size of ovaries in distal florets. In this study, we measured ovary sizes of proximal florets and distal floret F4. In the greenhouse experiment, ovary size of F4 was related to FFS (Fig. 7a, open symbols), implying that in this condition cultivars with more resource allocation for growth to distal florets presented higher levels of spike fertility. However, in the field experiments (in which the size of ovaries was larger than in the greenhouse), there was only a weak relationship (Fig. 7a, closed symbols). In both field and greenhouse conditions, the number of grains per spike was related to ovary sizes of distal florets at AN (Fig. 7b), suggesting that floret fertility was improved through the generation of more fertile distal florets. Distal florets with smaller ovaries were usually more prone to abortion, which in turn may have resulted in fewer grains being produced; this trend occurred in both environments (Fig. 7c, d) although it was statistically significant only in the greenhouse experiment (Fig. 7c).

## Discussion

In this study, we illustrated genotypic variation in floret development traits determining spike fertility, and presented relationships between them, in two contrasting environments (field and greenhouse). The analytical framework included three parameters in the dynamics of generation/degeneration of organs determining spike fertility: MFS, FFS, and GS. The MFS is the consequence of floret primordia initiation starting around TS stage and finishing approximately at GA; the FFS is the outcome of the floret primordia mortality/survival process occurring broadly from GA to AN; and GS is the result of grain set/abortion taking place in the ‘lag phase’ of roughly 7–10 days after AN to the onset of grain growth. Here, we not only quantified the outcomes of floret initiation, floret mortality/survival, and grain set/abortion in 30 modern cultivars (this analysis comprising for the first time such a large set of genotypes), but also tried, for the first time, to connect parts of these processes by describing the association between ovary size of distal florets with the number of fertile florets and grains. Interestingly, MFS, FFS, and GS exhibited not only large variation in both growth conditions and across all spikelet positions studied, but also displayed moderate levels of heritability. It has previously been reported that ovary size has high heritability (Komaki and Tsunewaki, 1981; Guo *et al.*, 2015). Understanding that these spike fertility traits have moderate heritabilities will be of use in further exploring their genetic basis in experimental populations.

In agreement with most agronomic literature, we found that floret initiation was far less relevant than the subsequent process of primordia degeneration to determine the number of fertile florets: FFS was closely associated with floret survival, but had a weak relationship with MFS. This association is consistent with previous work in wheat (Miralles *et al.*, 1998b; Gonzalez *et al.*, 2011a; Ferrante *et al.*, 2013b). Gonzalez *et al.* (2005) reported that a period of extended floret development, due to exposure to a short photoperiod during the stem elongation period, can increase the number of fertile florets at AN by improved floret survival while not influencing the maximum number of floret primordia initiated.

Furthermore, we also found that the post-AN process of grain set/abortion is also important for determining genotypic variation in grain number, although it was previously concluded that the number of grains per spike is mainly a consequence of floret initiation and degradation (fertile florets) (Kirby, 1988). Here we showed that the increase in GS was closely associated with improved grain survival. If spikelet positions along the spike are considered, it becomes clear that the central part of the spike dominates to produce fertile florets and to set grains (Rawson and Evans, 1970; Evans *et al.*, 1972; Pinthus and Millet, 1978; Millet, 1986). In our work, the relationship between GS and grain abortion after AN was maintained, whereas associations between GS and FFS were maintained in the greenhouse experiment but disappeared in apical and central spikelets (and were maintained only in basal spikelets, ‘basal’ here being the low central spikelets of the spike, not the extreme bottom spikelets) in the field experiment. These variable correlations indicate that spikelets at different positions of the spike possess variable sensitivities to growth conditions. Hence, the effects of spikelet positions along spikes should be considered in further studies of spike fertility.

Ovary size at AN is the result of floret initiation and degradation, and represents a possible predictor for grain setting (Guo *et al.*, 2015), so we consider it as the ‘connector’ between floret survival (pre-AN) and grain survival (post-AN). In this work, proximal florets (i.e. the first three florets from the base of a spikelet, F1, F2, and F3) did produce fertile florets and set grains in most cases. Ovary sizes of more distal florets (F4 and beyond) appeared to be a critical factor for grain setting and also effectively reflected the pre-AN floret development. In both environments, GS displayed positive correlations with ovary sizes of distal florets (≥F4), suggesting a critical role of photoassimilates partitioned to distal florets in regulating grain set (although environmental factors that are involved in anther and ovary abortion should not be neglected). In other words, improving floret fertility at AN may be irrelevant when more resources are not allocated to the growth of these florets, because the small florets, albeit fertile, would become abortive even if after pollination (post-AN grain abortion). This is in agreement with previous findings where access to assimilates plays a crucial role for spike fertility (Gonzalez *et al.*, 2011a; Guo and Schnurbusch, 2015). Interestingly, we only found a positive association between ovary size of F4 and FFS in the greenhouse condition. The fact that the development of distal florets is relatively sensitive to environments (Guo *et al.*, 2015) could be helpful in explaining the weak correlations found in the field. Besides the close correlation between ovary size and grain setting, one reason for highlighting the importance of ovary size is that the effects of some other factors such as assimilate allocation and anther size are reflected by ovary size. For example, anther size is closely associated with ovary size (Guo *et al.*, 2015). In addition, increasing FE (i.e. grain set per unit of spike dry weight at AN) is another way to improve grain setting. Slafer *et al.* (2015a) suggested two pathways to improve FE: (1) by increasing assimilate partitioning to floret primordia during pre-AN development, or (2) by reducing assimilate demand for floret development. The first option may lead to an increase in fertile florets without penalizing ovary size at AN (or to an increase in ovary size without penalizing the number of fertile florets); in option 2, the increase in number of fertile florets associated with higher FE would be achieved at the expense of reducing ovary size (because the amount of assimilates being consumed per developing primordium is decreased). Our data support option 1 because in the field we found an increase in spike dry weight that was associated with bigger ovary size (Guo *et al.*, 2015), which most likely was due to a longer duration (longer growth time, by days) of pre-AN phases. In a concurrent study, Elia *et al.* (2016) also found that differences in FE between modern cultivars did not reflect penalties in ovary size when the number of fertile florets was increased. It was also detected that the maximum number of floret primordia under field conditions was generally increased compared with greenhouse conditions, further suggesting that a longer duration from floret primordia initiation to GA stage increases floret primordia in the field (Guo *et al.*, 2015).

The observed non-grain setting even in F1 and F2 under ‘ideal conditions’ suggests other limiting factors for grain setting. For example, some stresses around meiosis (e.g. drought, B deficiency) can reduce grain set in these favoured positions, giving obvious spike ‘sterility’ despite no obvious growth limitations (Huang *et al.*, 2000; Ji *et al.*, 2010). Rawson and Evans (1970) found that F1 and F2 can inhibit grain formation in F3 and F4 even though the latter is perfectly competent to form grains. Clearly, spike fertility is a complex trait and displays strong genotypic variation; however, this study found ovary size to be a potentially promising factor in determining overall spike fertility.

## Supplementary data

Supplementary data are available at *JXB* online

Table S1. Marker information for all 30 cultivars used.

Table S2. Maximum number of floret primordia and fertile floret number in apical, central, and basal spikelets for individual genotypes of all the 30 cultivars in the greenhouse.

Table S3. Maximum number of floret primordia and fertile floret number per spikelet in apical, central, and basal spikelets for individual genotypes of all the 30 cultivars in the field.

Table S4. Final grain number per spikelet in apical, central, and basal spikelets and fruiting efficiency for individual genotypes of all the 30 cultivars in the greenhouse.

Table S5. Final grain number per spikelet in apical, central, and basal spikelets and fruiting efficiency for individual genotypes of all the 30 cultivars in the field.

Figure S1. Fruiting efficiency of 30 cultivars in the greenhouse and field.

Figure S2. Relationships between maximum number of floret primordia (spikelet^−1^), number of fertile florets (spikelet^−1^), number of grains (spikelet^−1^), and fruiting efficiency between greenhouse and field conditions.

Figure S3. Relationship between the maximum number of floret primordia and floret survival within apical, central, and basal spikelets under field and greenhouse conditions.

Figure S4. Relationship between the maximum number of floret primordia, fertile floret number, and floret abortion within apical, central, and basal spikelets under field and greenhouse conditions.

Figure S5. Relationship between the maximum number of fertile florets, number of grains, and grain abortion in apical, central, and basal spikelets under field and greenhouse conditions.

Supplementary Data

## Abbreviations:

AN: anthesis
F*x* ovary: ovary in the *x*th floret from the rachis
FE: fruiting efficiency
FFS: number of fertile florets per spikelet
GA: green anther
GS: number of grains per spikelet
MFS: maximum number of floret primordia per spikelet
PM: physiological maturity
TS: terminal spikelet.
