# Supplementary material for: Genotypic variation in spike fertility traits and ovary size as determinants of floret and grain survival rate in wheat
Source: J Exp Bot. 2016 Jun 8;67(14):4221–30. doi: 10.1093/jxb/erw200 (PMC5301927; doi:10.1093/jxb/erw200)
Supplement: Supplementary Data [file supp_67_14_4221__index.html]

Genotypic variation in spike fertility traits and ovary size as determinants of floret and grain survival rate in wheat — Genotypic variation in spike fertility traits and ovary size as determinants of floret and grain survival rate in wheat — Supplementary Data 

# Genotypic variation in spike fertility traits and ovary size as determinants of floret and grain survival rate in wheat

## Supplementary Data

Data files

- Supplementary\_Tables\_S1\_S5\_Figures\_S1\_S5.pdf - Supplementary Data
