# Supplementary material for: Genotypic variation in spike fertility traits and ovary size as determinants of floret and grain survival rate in wheat
Source: J Exp Bot. 2016 Jun 8;67(14):4221–30. doi: 10.1093/jxb/erw200 (PMC5301927; doi:10.1093/jxb/erw200)
Supplement: Supplementary Data [file supp_erw200_Supplementary_Tables_S1_S5_Figures_S1_S5.pdf]

Supplementary materials

Table S1. Marker information for all 30 cultivars used.

Running number	Cultivar name	*Rht-B1* (+/-)*	*Rht-D1* (+/-)*	*Ppd-D1* (+/-)§
1	Bussard	-	-	-
2	Tukan	-	+	+
3	Tulsa	+	-	+
4	Türkis	-	+	+
5	Winnetou	-	-	+
6	Zentos	-	-	+
7	Zobel	-	+	+
8	Cliff	-	+	+
9	Dream	-	-	+
10	Florett	-	+	+
11	History	-	-	+
12	Lindos	-	-	+
13	Julius	-	+	+
14	Exotic	-	+	-
15	Andalou	-	+	-
16	Loch	-	+	+
17	Acienda	-	+	-
18	Aguila	-	+	-
19	Alcazar	-	+	+
20	Allister	-	+	+
21	Arack	-	+	+
22	Arobase	-	+	+
23	Astuce	-	+	+
24	Aubusson	-	+	+
25	Autan	-	+	-
26	Azimut	-	+	-
27	Azzuro	-	+	+
28	Balance	-	+	+
29	Baltimor	-	+	+
30	Bastide	-	+	+

*(+) indicates the presence of a semi-dwarfing allele
§(+) indicates the presence of the photoperiod-sensitive allele at the *Ppd-D1* locus.

Table S2. Maximum number of floret primordia and fertile floret number in apical, central and basal spikelets for individual genotype of all the 30 cultivars in the greenhouse (mean ± standard deviation).

Genotype	Apical max	Central max	Basal max	Apical fertile	Central fertile	Basal fertile
Bussard	9.00 ± 1.00	10.00 ± 1.00	9.33 ± 0.58	3.33 ± 0.58	4.33 ± 0.58	4.00 ± 1.00
Tukan	8.33 ± 0.58	9.67 ± 0.58	9.00 ± 0.00	4.00 ± 0.00	4.67 ± 0.58	4.67 ± 0.58
Tulsa	8.50 ± 0.71	10.00 ± 0.00	10.00 ± 0.00	4.00 ± 1.00	4.67 ± 0.58	5.00 ± 1.00
Türkis	9.33 ± 1.15	9.67 ± 0.58	9.67 ± 0.58	4.00 ± 0.00	4.33 ± 0.58	4.33 ± 0.58
Winnetou	9.00 ± 1.00	10.33 ± 0.58	10.00 ± 0.00	4.33 ± 1.15	6.00 ± 0.00	5.67 ± 0.58
Zentos	8.67 ± 0.58	10.33 ± 0.58	9.67 ± 0.58	3.33 ± 0.58	5.33 ± 0.58	5.00 ± 0.00
Zobel	9.67 ± 0.58	10.67 ± 0.58	10.00 ± 1.00	4.00 ± 0.00	4.67 ± 0.58	4.00 ± 0.00
Cliff	9.00 ± 0.00	9.00 ± 0.00	11.00 ± 0.00	4.00 ± 0.00	5.33 ± 0.58	5.33 ± 0.58
Dream	8.67 ± 0.58	10.00 ± 0.00	9.67 ± 0.58	4.00 ± 1.41	5.50 ± 0.71	5.00 ± 0.00
Florett	8.33 ± 0.58	10.00 ± 1.00	9.00 ± 1.00	4.00 ± 0.00	5.33 ± 0.58	5.00 ± 0.00
History	9.33 ± 0.58	9.67 ± 0.58	9.00 ± 0.00	4.00 ± 0.00	5.00 ± 0.00	5.67 ± 0.58
Lindos	8.33 ± 0.58	10.00 ± 0.00	9.00 ± 0.00	4.00 ± 0.00	5.00 ± 0.00	5.00 ± 0.00
Julius	9.00 ± 1.00	9.67 ± 0.58	10.00 ± 0.00	3.00 ± 0.00	4.00 ± 0.00	4.00 ± 0.00
Exotic	7.67 ± 1.15	8.33 ± 0.58	8.00 ± 1.00	3.33 ± 0.58	3.67 ± 0.58	3.33 ± 0.58
Andalou	9.00 ± 0.00	10.00 ± 0.00	9.00 ± 0.00	3.00 ± 0.00	4.00 ± 0.00	3.67 ± 0.58
Loch 3754						
Adlon	8.33 ± 0.58	10.00 ± 0.00	9.33 ± 0.58	5.33 ± 0.58	6.00 ± 0.00	5.33 ± 0.58
Acienda	9.00 ± 0.00	9.33 ± 1.15	9.00 ± 1.00	4.33 ± 0.58	5.00 ± 0.00	4.33 ± 0.58
Aguila	8.33 ± 1.15	9.00 ± 0.00	9.00 ± 0.00	3.67 ± 0.58	4.67 ± 0.58	4.00 ± 0.00
Alcasar	8.67 ± 0.58	9.67 ± 0.58	9.67 ± 0.58	4.00 ± 0.00	5.00 ± 0.00	4.67 ± 0.58
Allister	9.00 ± 0.00	9.67 ± 0.58	9.00 ± 1.00	3.00 ± 0.00	4.00 ± 0.00	4.00 ± 0.00
Arack	8.50 ± 0.71	9.50 ± 0.71	10.00 ± 0.00	4.00 ± 0.00	5.00 ± 0.00	5.00 ± 0.00
Arobase	9.33 ± 1.15	9.67 ± 0.58	8.67 ± 0.58	5.00 ± 0.00	5.00 ± 0.00	5.00 ± 0.00
Astuce	8.67 ± 0.58	9.33 ± 0.58	9.67 ± 0.58	3.33 ± 0.58	4.00 ± 0.00	4.00 ± 0.00
Abusson	9.00 ± 1.00	10.00 ± 1.00	10.00 ± 0.00	4.33 ± 0.58	4.33 ± 0.58	4.33 ± 0.58
Autan	8.33 ± 0.58	8.67 ± 0.58	8.67 ± 0.58	3.33 ± 0.58	3.67 ± 0.58	3.67 ± 0.58
Azimut	9.00 ± 0.00	9.67 ± 0.58	10.00 ± 0.00	4.00 ± 0.00	4.33 ± 0.58	4.33 ± 0.58
Azzuro	9.00 ± 0.00	9.50 ± 0.71	10.00 ± 0.00	3.67 ± 0.58	4.33 ± 0.58	4.00 ± 0.00
Balance	9.00 ± 0.00	9.00 ± 0.00	11.00 ± 0.00	4.00 ± 0.00	5.00 ± 0.00	5.00 ± 0.00
Baltimor	9.00 ± 0.00	9.67 ± 0.58	9.00 ± 0.00	3.33 ± 0.58	4.67 ± 0.58	4.33 ± 0.58
Bastide	9.33 ± 0.58	9.67 ± 0.58	9.33 ± 0.58	5.00 ± 0.00	5.33 ± 0.58	5.00 ± 0.00

Table S3. Maximum number of floret primordia and fertile floret number per spikelet in apical, central and basal spikelets for individual genotype of all the 30 cultivars in the field (mean ± standard deviation).

Genotype	Apical max	Central max	Basal max	Apical fertile	Central fertile	Basal fertile
Bussard	9.33 ± 0.58	10.33 ± 0.58	10.33 ± 0.58	4.00 ± 0.00	5.33 ± 0.58	5.33 ± 0.58
Tukan	10.00 ± 0.00	10.00 ± 0.00	10.00 ± 0.00	3.67 ± 0.58	4.67 ± 0.58	4.67 ± 0.58
Tulsa	11.00 ± 0.00	11.00 ± 0.00	10.00 ± 1.00	4.33 ± 0.58	5.33 ± 0.58	5.33 ± 0.58
Türkis	9.67 ± 0.58	10.33 ± 0.58	10.00 ± 1.00	4.00 ± 0.00	4.33 ± 0.58	4.33 ± 0.58
Winnetou	9.67 ± 0.58	10.00 ± 0.00	10.00 ± 0.00	4.00 ± 0.00	4.33 ± 0.58	4.33 ± 0.58
Zentos	9.67 ± 0.58	10.33 ± 0.58	10.00 ± 0.00	3.67 ± 0.58	5.00 ± 0.00	5.00 ± 0.00
Zobel	10.50 ± 0.71	11.50 ± 0.71	10.00 ± 0.00	3.33 ± 0.58	5.00 ± 0.00	5.00 ± 0.00
Cliff	10.00 ± 1.00	11.00 ± 1.00	11.00 ± 1.00	4.00 ± 0.00	5.00 ± 0.00	4.67 ± 0.58
Dream	10.33 ± 0.58	10.33 ± 0.58	10.33 ± 0.58	3.33 ± 0.58	4.33 ± 0.58	5.00 ± 0.00
Florett	10.00 ± 0.00	10.67 ± 0.58	10.67 ± 0.58	3.67 ± 0.58	4.67 ± 0.58	4.33 ± 0.58
History	10.00 ± 0.00	11.00 ± 0.00	9.67 ± 0.58	4.33 ± 0.58	5.67 ± 0.58	5.00 ± 1.00
Lindos	10.67 ± 1.15	11.67 ± 0.58	11.33 ± 0.58	3.67 ± 0.58	5.33 ± 0.58	5.00 ± 0.00
Julius	10.00 ± 1.00	10.00 ± 1.00	10.00 ± 0.00	3.67 ± 0.58	4.33 ± 0.58	4.33 ± 0.58
Exotic	9.33 ± 0.58	9.67 ± 0.58	9.33 ± 0.58	4.67 ± 0.58	5.00 ± 0.00	5.00 ± 0.00
Andalou	9.67 ± 0.58	10.00 ± 1.00	9.67 ± 1.53	3.33 ± 0.58	4.00 ± 0.00	4.00 ± 0.00
Loch 3754						
Adlon	10.00 ± 0.00	10.50 ± 0.58	9.50 ± 0.58	4.00 ± 0.00	5.00 ± 0.00	4.67 ± 0.58
Acienda	9.67 ± 0.58	10.33 ± 0.58	10.33 ± 0.58	4.67 ± 0.58	5.00 ± 1.00	4.67 ± 0.58
Aguila	9.67 ± 0.58	11.00 ± 1.00	10.67 ± 0.58	4.33 ± 0.58	5.33 ± 0.58	5.33 ± 0.58
Alcasar	11.00 ± 0.00	11.00 ± 0.00	11.00 ± 0.00	3.33 ± 0.58	5.00 ± 0.00	4.33 ± 0.58
Allister	10.00 ± 0.00	11.00 ± 0.00	10.67 ± 0.58	4.33 ± 0.58	4.67 ± 0.58	4.67 ± 0.58
Arack	10.67 ± 0.58	11.00 ± 0.00	10.00 ± 0.00	5.33 ± 0.58	6.00 ± 0.00	6.00 ± 0.00
Arobase	11.00 ± 0.00	11.67 ± 0.58	11.00 ± 0.00	4.33 ± 0.58	5.33 ± 0.58	5.33 ± 0.58
Astuce	9.67 ± 0.58	10.33 ± 1.15	10.33 ± 0.58	4.33 ± 0.58	5.67 ± 0.58	5.67 ± 0.58
Abusson	9.67 ± 0.58	11.00 ± 0.00	11.00 ± 0.00	4.00 ± 0.00	4.67 ± 0.58	4.67 ± 0.58
Autan	9.33 ± 0.58	10.33 ± 0.58	9.67 ± 0.58	3.33 ± 0.58	4.33 ± 0.58	4.33 ± 0.58
Azimut	9.67 ± 0.58	10.67 ± 0.58	10.33 ± 0.58	4.33 ± 0.58	5.67 ± 0.58	5.67 ± 0.58
Azzuro	9.33 ± 0.58	10.00 ± 1.00	11.00 ± 0.00	4.67 ± 0.58	5.67 ± 0.58	5.00 ± 0.00
Balance	10.00 ± 0.00	11.00 ± 0.00	10.67 ± 0.58	4.67 ± 0.58	5.67 ± 0.58	5.67 ± 0.58
Baltimor	9.00 ± 0.00	10.33 ± 0.58	10.33 ± 0.58	4.00 ± 0.00	5.00 ± 0.00	5.00 ± 0.00
Bastide	10.00 ± 0.00	10.33 ± 0.58	10.00 ± 1.00	4.33 ± 0.58	5.33 ± 0.58	5.00 ± 1.00

Table S4. Final grain number per spikelet in apical, central and basal spikelets and fruiting efficiency for individual genotype of all the 30 cultivars in the greenhouse (mean ± standard deviation).

Genotype	apical final grain	central final grain	basal final grain	fruiting efficiency
Bussard	3.00 ± 0.63	4.17 ± 0.75	4.17 ± 0.75	14.69 ± 2.02
Tukan	2.33 ± 0.82	3.33 ± 1.21	3.67 ± 1.51	15.74 ± 1.34
Tulsa	2.67 ± 0.52	3.17 ± 1.47	4.33 ± 1.03	25.80 ± 4.08
Türkis	2.50 ± 1.22	3.33 ± 1.51	2.50 ± 1.05	16.82 ± 0.99
Winnetou	2.33 ± 1.03	4.00 ± 0.89	4.50 ± 1.05	17.44 ± 1.25
Zentos	3.33 ± 0.52	4.00 ± 0.89	4.00 ± 0.63	15.68 ± 1.53
Zobel	3.67 ± 0.58	5.00 ± 1.00	4.33 ± 0.58	17.34 ± 1.47
Cliff	3.33 ± 0.82	4.50 ± 0.84	3.67 ± 1.51	19.74 ± 0.76
Dream	2.67 ± 0.52	3.67 ± 0.52	3.67 ± 0.52	21.60 ± 2.43
Florett	2.67 ± 0.52	3.14 ± 0.75	2.57 ± 0.82	19.03 ± 1.75
History	3.00 ± 1.22	4.20 ± 1.30	2.75 ± 1.71	17.08 ± 1.36
Lindos	2.40 ± 0.89	3.80 ± 1.10	2.20 ± 0.84	18.79 ± 0.78
Julius	2.25 ± 0.96	3.33 ± 0.58	2.50 ± 0.58	18.38 ± 1.34
Exotic	3.33 ± 0.82	4.33 ± 0.82	3.67 ± 1.03	15.33 ± 2.02
Andalou	2.86 ± 0.63	4.00 ± 0.75	3.71 ± 0.84	16.23 ± 1.23
Loch 3754				
Adlon	3.00 ± 0.89	4.00 ± 0.89	2.67 ± 1.37	20.56 ± 1.05
Acienda	3.33 ± 0.82	3.33 ± 0.82	3.17 ± 0.75	21.76 ± 2.78
Aguila	3.17 ± 0.98	3.67 ± 1.21	4.00 ± 1.41	20.38 ± 1.87
Alcasar	2.43 ± 0.52	3.43 ± 1.22	3.71 ± 1.21	20.11 ± 2.30
Allister	3.50 ± 0.55	4.00 ± 0.63	3.83 ± 1.17	18.57 ± 0.70
Arack	3.17 ± 0.75	4.50 ± 1.05	4.33 ± 1.37	15.76 ± 2.32
Arobase	4.33 ± 0.82	4.17 ± 0.98	4.50 ± 1.05	20.46 ± 1.57
Astuce	2.80 ± 0.84	3.60 ± 0.89	3.40 ± 1.14	19.05 ± 1.54
Abusson	3.00 ± 0.89	4.43 ± 1.03	4.14 ± 0.75	16.78 ± 1.02
Autan	3.57 ± 1.10	4.14 ± 0.52	3.43 ± 0.55	14.54 ± 0.78
Azimut	3.43 ± 0.84	4.00 ± 0.41	3.57 ± 0.75	22.88 ± 2.71
Azzuro	2.86 ± 1.03	3.86 ± 1.33	4.43 ± 0.84	17.10 ± 0.69
Balance	3.00 ± 1.41	3.67 ± 1.03	3.67 ± 1.37	17.83 ± 1.08
Baltimor	3.00 ± 0.89	4.00 ± 0.89	3.67 ± 0.82	18.06 ± 0.83
Bastide	3.33 ± 1.03	4.17 ± 1.17	4.00 ± 1.26	17.23 ± 0.70

Table S5. Final grain number per spikelet in apical, central and basal spikelets and fruiting efficiency for individual genotype of all the 30 cultivars in the field (mean ± standard deviation).

Genotype	apical final grain	central final grain	basal final grain	fruiting efficiency
Bussard	1.00 ± 1.26	3.17 ± 0.75	3.83 ± 0.41	15.10 ± 1.69
Tukan	3.33 ± 0.82	4.00 ± 0.00	4.00 ± 0.00	17.02 ± 2.60
Tulsa	3.33 ± 0.52	3.67 ± 0.52	3.50 ± 1.05	15.89 ± 5.15
Türkis	2.83 ± 1.47	4.17 ± 0.41	4.33 ± 0.52	20.18 ± 2.90
Winnetou	1.83 ± 1.72	4.67 ± 0.52	4.67 ± 0.52	20.63 ± 5.54
Zentos	1.83 ± 0.98	3.67 ± 0.52	3.83 ± 0.75	16.65 ± 5.37
Zobel	3.17 ± 0.41	3.83 ± 0.41	3.50 ± 1.22	17.58 ± 1.54
Cliff	2.67 ± 0.52	4.17 ± 0.75	3.50 ± 1.05	16.67 ± 3.46
Dream	0.50 ± 1.00	3.50 ± 0.58	3.25 ± 0.96	16.25 ± 5.42
Florett	2.67 ± 0.52	3.67 ± 0.52	3.33 ± 0.82	17.49 ± 3.67
History	3.00 ± 0.63	4.17 ± 0.41	4.17 ± 0.75	15.02 ± 5.92
Lindos	2.33 ± 0.82	4.00 ± 0.89	3.33 ± 0.82	28.93 ± 13.34
Julius	2.67 ± 0.52	3.50 ± 0.55	3.67 ± 0.52	26.80 ± 13.58
Exotic	3.17 ± 0.41	3.33 ± 0.52	3.17 ± 0.41	15.17 ± 5.59
Andalou	3.00 ± 0.63	3.83 ± 0.41	3.67 ± 0.52	15.84 ± 5.66
Loch 3754				
Adlon	3.83 ± 0.41	4.50 ± 0.55	4.33 ± 1.21	19.78 ± 9.62
Acienda	3.67 ± 0.52	4.50 ± 0.55	3.67 ± 0.52	20.03 ± 7.67
Aguila	3.17 ± 0.75	4.00 ± 0.00	3.50 ± 0.55	18.19 ± 3.97
Alcasar	3.17 ± 0.75	4.33 ± 0.82	4.33 ± 0.52	17.76 ± 3.19
Allister	2.50 ± 0.55	3.33 ± 0.52	3.50 ± 0.55	18.70 ± 5.36
Arack	3.33 ± 0.52	3.83 ± 0.75	3.83 ± 0.41	15.01 ± 3.81
Arobase	4.33 ± 0.52	4.67 ± 0.52	4.17 ± 0.41	18.95 ± 5.68
Astuce	3.00 ± 0.00	4.17 ± 0.41	4.00 ± 0.00	14.29 ± 5.78
Abusson	3.17 ± 0.41	4.17 ± 0.41	3.50 ± 0.55	17.75 ± 6.46
Autan	3.00 ± 0.00	3.33 ± 0.52	2.83 ± 0.75	14.36 ± 2.33
Azimut	3.33 ± 0.52	4.17 ± 0.41	3.83 ± 0.41	19.45 ± 7.31
Azzuro	3.17 ± 0.41	3.50 ± 0.84	3.83 ± 0.41	19.38 ± 6.73
Balance	3.33 ± 0.82	4.50 ± 0.55	4.33 ± 0.52	15.27 ± 1.07
Baltimor	2.83 ± 0.41	4.00 ± 0.00	4.00 ± 0.00	17.75 ± 2.89
Bastide	4.17 ± 1.17	5.00 ± 0.00	4.67 ± 0.52	16.93 ± 1.83

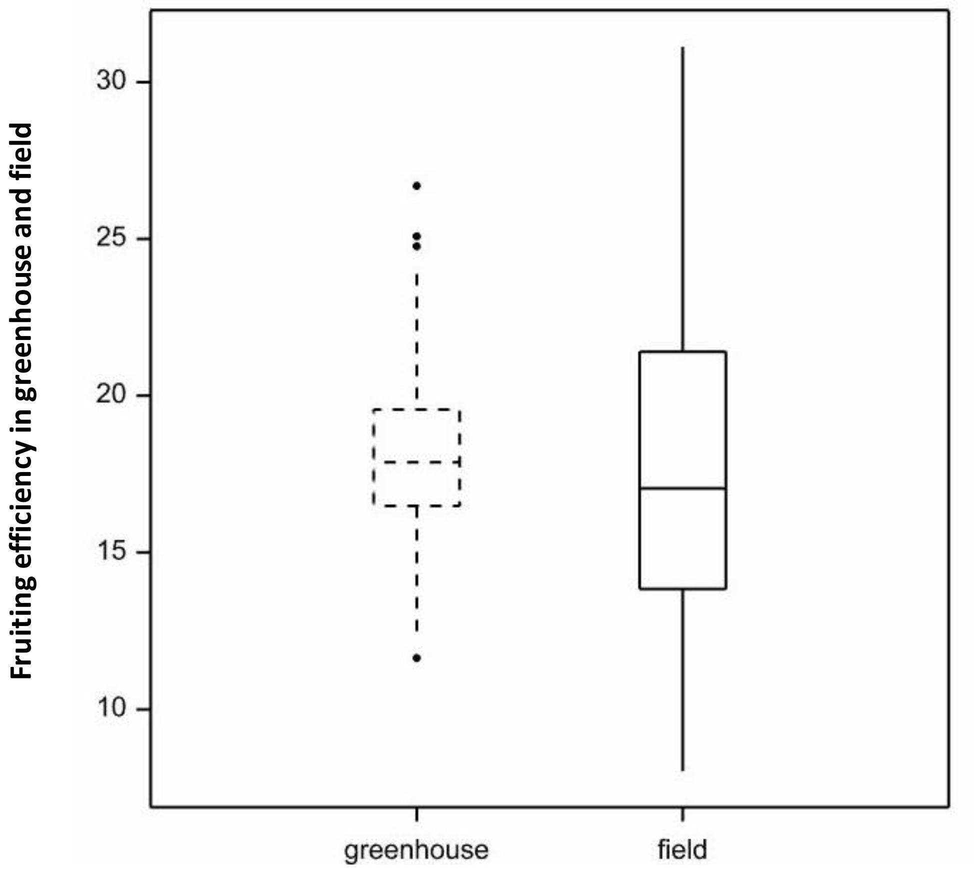

Fig. S1. Fruiting efficiency for the 30 cultivars in the greenhouse and field.

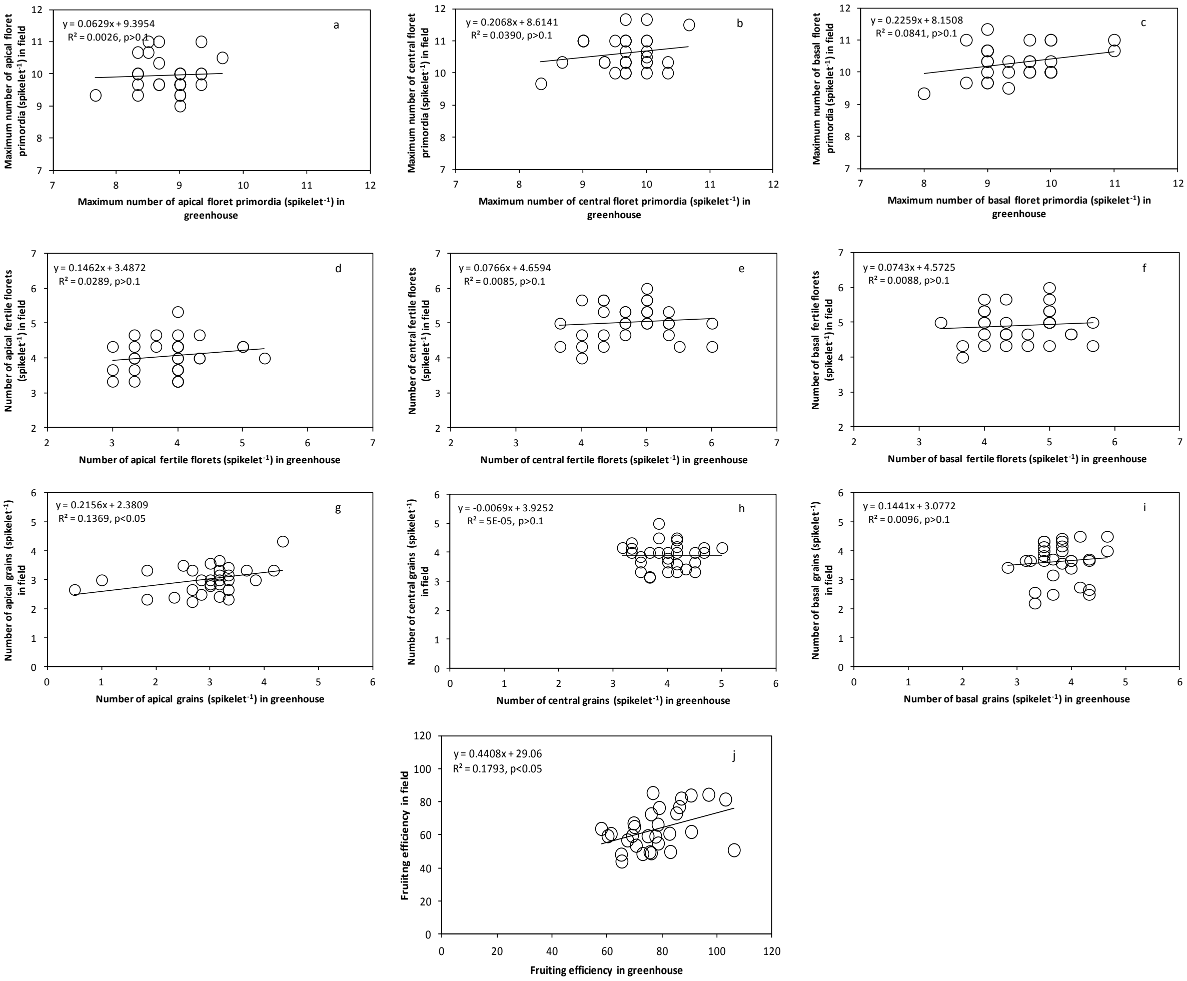

Fig. S2. Relationships of maximum number of floret primordia (spikelet^{-1}), number of fertile florets (spikelet^{-1}), number of grains (spikelet^{-1}) and fruiting efficiency between greenhouse and field.

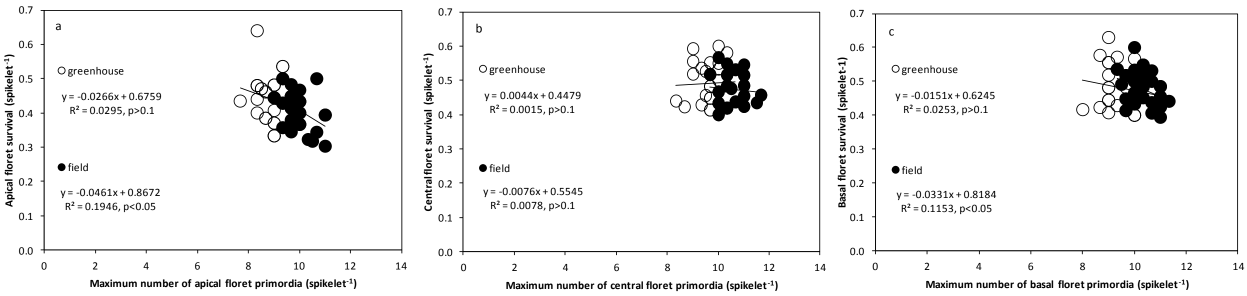

Fig. S3. Relationship between maximum number of floret primordia and floret survival within spikelet in apical, central and basal parts of spike under field and greenhouse conditions.

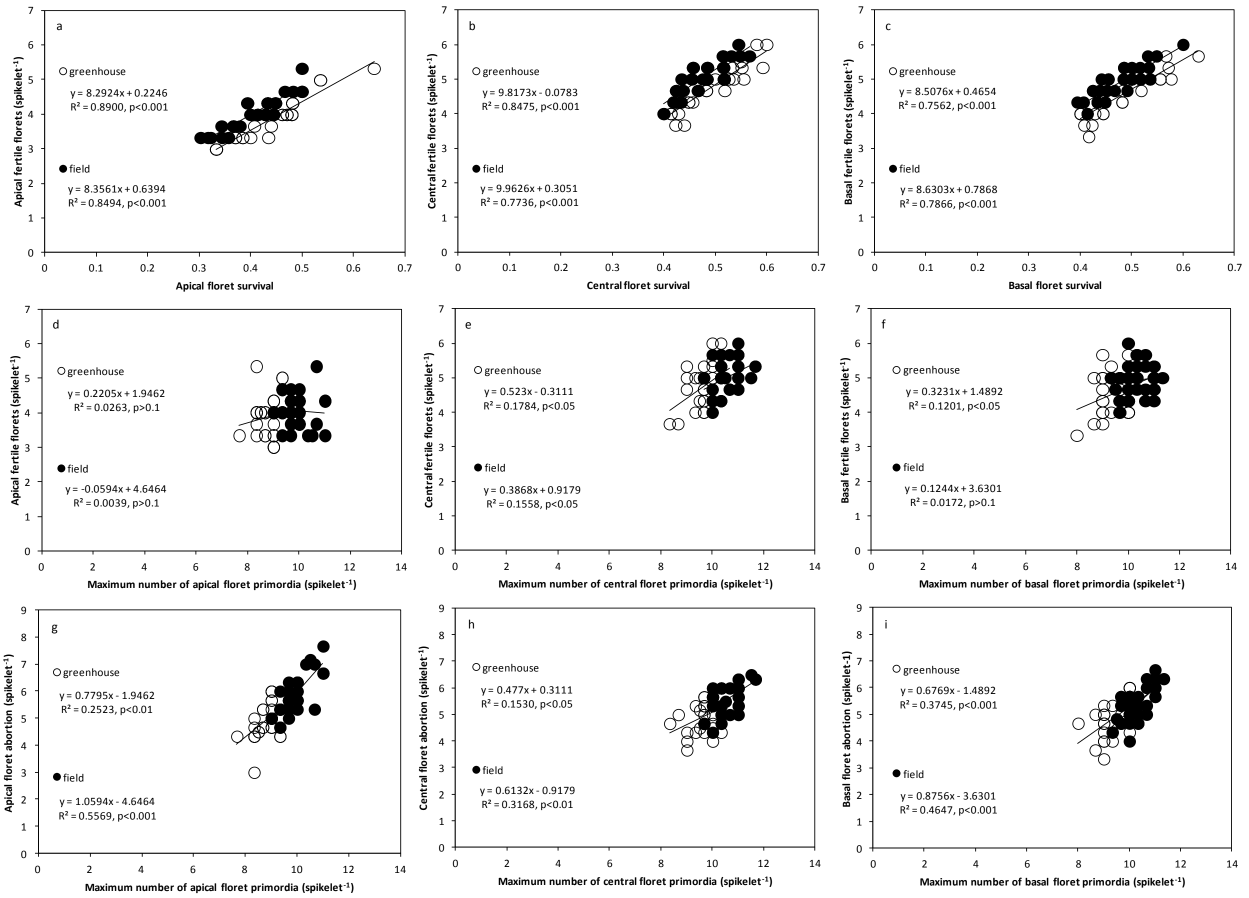

Fig. S4. Relationship between maximum number of floret primordia, fertile florets number and floret abortion within spikelet in apical, central and basal spikelets under field and greenhouse conditions.

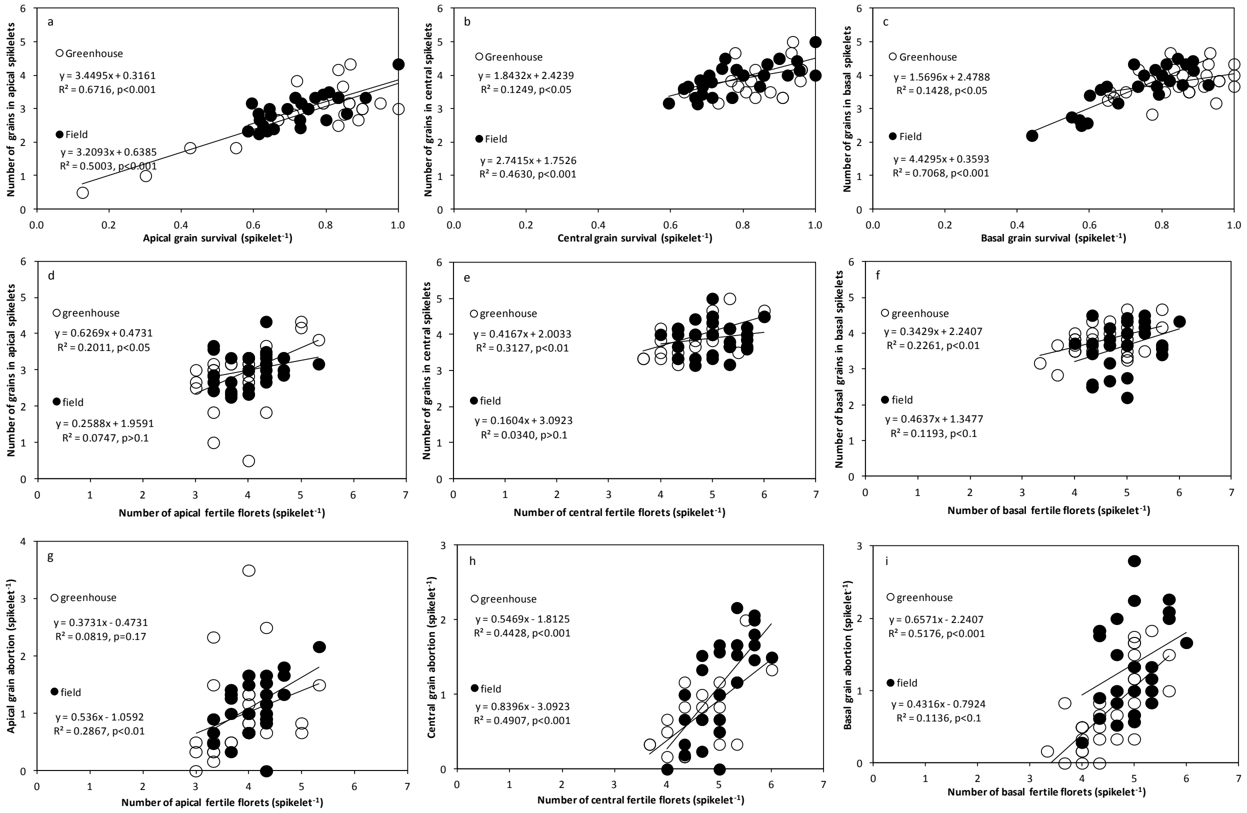

Fig. S5. Relationship between maximum number of fertile florets, number of grains and grain abortion per spikelet in apical, central and basal positions of spike under field and greenhouse conditions.
